# Variable Neural Contributions to Explicit and Implicit Learning During Visuomotor Adaptation

**DOI:** 10.3389/fnins.2018.00610

**Published:** 2018-09-18

**Authors:** Sook-Lei Liew, Tziporah Thompson, Joel Ramirez, Peter A. Butcher, Jordan A. Taylor, Pablo A. Celnik

**Affiliations:** ^1^National Institute of Neurological Disorders and Stroke, National Institutes of Health, Bethesda, MD, United States; ^2^Division of Occupational Science and Occupational Therapy, Division of Biokinesiology and Physical Therapy, Department of Neuroogy, University of Southern California, Los Angeles, CA, United States; ^3^Department of Physical Medicine and Rehabilitation, Johns Hopkins University School of Medicine, Baltimore, MD, United States; ^4^Department of Psychology and Princeton Neuroscience Institute, Princeton University, Princeton, NJ, United States

**Keywords:** visuomotor adaptation-learning, tDCS, explicit learning, implicit learning, cerebellum, dorsolateral prefrontal cortex (DLPFC), context-dependent learning

## Abstract

We routinely make fine motor adjustments to maintain optimal motor performance. These adaptations have been attributed to both implicit, error-based mechanisms, and explicit, strategy-based mechanisms. However, little is known about the neural basis of implicit vs. explicit learning. Here, we aimed to use anodal transcranial direct current stimulation (tDCS) to probe the relationship between different brain regions and learning mechanisms during a visuomotor adaptation task in humans. We hypothesized that anodal tDCS over the cerebellum (CB) should increase implicit learning while anodal tDCS over the dorsolateral prefrontal cortex (dlPFC), a region associated with higher-level cognition, should facilitate explicit learning. Using a horizontal visuomotor adaptation task that measures explicit/implicit contributions to learning (Taylor et al., 2014), we found that dlPFC stimulation significantly improved performance compared to the other groups, and weakly increased explicit learning. However, CB stimulation had no effects on either target error or implicit learning. Previous work showed variable CB stimulation effects only on a vertical visuomotor adaptation task (Jalali et al., 2017), so in Experiment 2, we conducted the same study using a vertical context to see if we could find effects of CB stimulation. We found only weak effects of CB stimulation on target error and implicit learning, and now the dlPFC effect did not replicate. To resolve this discrepancy, in Experiment 3, we examined the effect of context (vertical vs. horizontal) on implicit and explicit contributions and found that individuals performed significantly worse and used greater implicit learning in the vertical screen condition compared to the horizontal screen condition. Across all experiments, however, there was high inter-individual variability, with strong influences of a few individuals, suggesting that these effects are not consistent across individuals. Overall, this work provides preliminary support for the idea that different neural regions can be engaged to improve visuomotor adaptation, but shows that each region's effects are highly context-dependent and not clearly dissociable from one another. This holds implications especially in neurorehabilitation, where an intact neural region could be engaged to potentially compensate if another region is impaired. Future work should examine factors influencing interindividual variability during these processes.

## Introduction

Motor actions are rapidly and continuously adjusted to accommodate changes in our bodies or our environment (Körding and Wolpert, 2006; Shadmehr et al., 2010). The visuomotor rotation task, where participants learn a new visuomotor mapping between movements of the hand and visual feedback, has served as a model paradigm to study this aspect of motor learning (Cunningham, 1989; Pine et al., 1996; Krakauer, 2009). Here, individuals are asked to make reaching movements to a target in the presence of a perturbation (e.g., 45° clockwise rotation), and learn to counteract this perturbation by reaching in opposite direction (e.g., 45° in counterclockwise direction). This type of learning is thought to rely on the formation of an internal model from sensorimotor prediction errors, based on the difference between the intended movement and visual feedback (Wolpert and Miall, 1996; Körding and Wolpert, 2004).

Researchers have proposed that explicit strategies, in which individuals attempt to overcome the rotation by consciously altering their aiming direction, may also play a critical role during visuomotor adaptation tasks (Mazzoni and Krakauer, 2006; Heuer and Hegele, 2008; Hegele and Heuer, 2010; Taylor et al., 2010, 2014; Benson et al., 2011; Taylor and Ivry, 2011, 2012). By incorporating a self-reported aiming direction into a classic visuomotor adaptation paradigm, Taylor et al. (2014) have shown the role of both explicit strategy and implicit error-based components as distinct mechanisms underlying learning adaptation tasks. Importantly, they show different conditions can influence the engagement of implicit and explicit mechanisms. For instance, their results show that feedback type (online vs. endpoint) affects the amount of explicit vs. implicit learning used. Specifically, they showed that online feedback, in which the cursor is visible throughout the movement, biased individuals toward a greater implicit contribution, while endpoint feedback, in which the cursor is shown only at its final location at the end of the movement, biased individuals toward a greater explicit contribution (Taylor et al., 2014). This suggests that other conditions may also affect explicit vs. implicit learning. However, it is unclear whether and how explicit and implicit components interact during learning, and what the neural bases of these components might be.

One way to better understand these interactions is by identifying the neural substrates responsible for implicit and explicit processes and examining whether they are distinct from one another. Excitatory stimulation, through anodal transcranial direct current stimulation (tDCS), is a noninvasive way to probe a neural region's involvement during a motor task (Nitsche et al., 2003; Reis and Fritsch, 2011). Importantly, as tDCS delivers steady low-amplitude current to the brain via surface electrodes, the physical sensations of tDCS are typically imperceptible, except during the initial 30 s during which the current ramps up. Because of this, stimulation can be provided while participants complete a task, such as visuomotor adaptation, without being disturbed by physical sensations from the stimulation. In addition, participants often cannot distinguish active tDCS from sham tDCS, making it a good noninvasive brain stimulation option for studying the neural effects of a region on behavior compared to a control sham condition (Gandiga et al., 2006). These reasons make tDCS more useful for the current study compared to other, more direct neuromodulation methods, such as transcranial magnetic stimulation (TMS), which provide stronger and more spatially-specific stimulation but also create physical sensations (auditory, somatosensory) during stimulation that could be distracting during the task and make it more difficult to provide a sham control condition.

Previously, research has shown that patients with cerebellar degeneration have difficulty with visuomotor adaptation tasks (Maschke et al., 2004; Morton and Bastian, 2006; Rabe et al., 2009) and that anodal excitatory tDCS over the cerebellum facilitates performance on these tasks (Galea et al., 2011; Block and Celnik, 2013; Hardwick and Celnik, 2014). However, more recent work has shown inconsistent results of cerebellar stimulation. Thus, we aimed to examine whether the implicit learning component of adaptation was reliably increased during anodal tDCS over the cerebellum (CB). While less is known about the explicit component of visuomotor adaptation, findings from cognitive neuroscience suggest the left dorsolateral prefrontal cortex (dlPFC) may be involved in strategy-based learning. As a key region involved in problem solving and fluid intelligence (Waltz et al., 1999; Kroger et al., 2002; Santarnecchi et al., 2013), the dlPFC has also been implicated in working memory specifically in relation to motor learning (Pascual-Leone et al., 1996; Fregni et al., 2005; Anguera et al., 2010). Thus, we also predicted that anodal tDCS over the left dlPFC should enhance explicit mechanisms of learning. To provide a baseline for comparison, we also had a group of individuals who received sham stimulation (SHAM).

Here, we performed three experiments. The first experiment lent support to our hypothesis that anodal dlPFC stimulation enhances performance on a visuomotor adaptation task, through a weak increase in explicit learning. In the second experiment, we found only a weak effect of CB stimulation on visuomotor adaptation compared to sham, and a trend toward CB stimulation increasing implicit learning. However, here stimulation of the dlPFC did not enhance overall performance, as it did in Experiment 1. Taken together, CB stimulation seems to have mild effects on implicit learning while dlPFC stimulation impacts explicit learning. However, these results are weak and highly variable across individuals and conditions. Indeed, individual subject analyses reveal that effects are primarily driven by poor performers in the SHAM group. The first two studies also suggest that the mechanisms used (explicit vs. implicit processes) are highly context-dependent and that there is large interindividual variability between participants within each group. In the third experiment, we thus examined how the physical context (e.g., vertical vs. horizontal orientation of the feedback screen) affects the ratio of explicit to implicit mechanisms involved in learning, and show a strong context-dependent effect on visuomotor adaptation.

## Materials and methods

### Participants

We recruited 111 individuals for this series of three experiments. Three participants were excluded due to technical difficulties or difficulty understanding the instructions, resulting in 108 participants [*n* = 48 for Experiment 1; *n* = 30 for Experiment 2; *n* = 30 for Experiment 3 (which were compared with participants from Experiment 2, explained below)]. Within each experiment, an outlier detection was run (see also Methods: Statistical Analyses), resulting in *n* = 46 for Experiment 1 (CB = 16, PFC = 15, SHAM = 15), *n* = 29 for Experiment 2 (CB = 10, PFC = 10, SHAM = 9), and *n* = 29 for Experiment 3 (CB = 10, PFC = 10, SHAM = 9). All participants were healthy, right-handed, and had not previously participated in a visuomotor adaptation study. This study was carried out in accordance with the recommendations of the Institutional Review Board (IRB) at Johns Hopkins University School of Medicine. The protocol was approved by the Johns Hopkins University School of Medicine IRB. All subjects gave written informed consent in accordance with the 1964 Declaration of Helsinki.

### Sample size and power calculation

We based our power calculation and sample size determination on data from a previous, similar study (Galea et al., 2011), which examined cerebellar tDCS effects on visuomotor adaptation compared to two other stimulation groups (SHAM, primary motor cortex). We used the mean error group differences during the first adaptation block from the second experiment, which compared the effects of cerebellar, M1, and SHAM stimulation on visuomotor adaptation, and which had an effect size of approximately f = 0.7. Using a one-way ANOVA with three groups, and assuming an α = 0.05 and power = 0.80, the suggested sample size was *n* = 8 per group. We therefore aimed to collect a minimum of *n* = 10 per group, and after outlier detection, this yielded a minimum of *n* = 9 per group, with a range of *n* = 9–16 per group depending on the experiment. Although small, this minimum group size was in line with the samples used in Galea et al. (2011) (group sizes of *n* = 8–10 across all experiments), which used a very similar paradigm and demonstrated strongly significant results.

### Experimental apparatus

**Figure 1:**
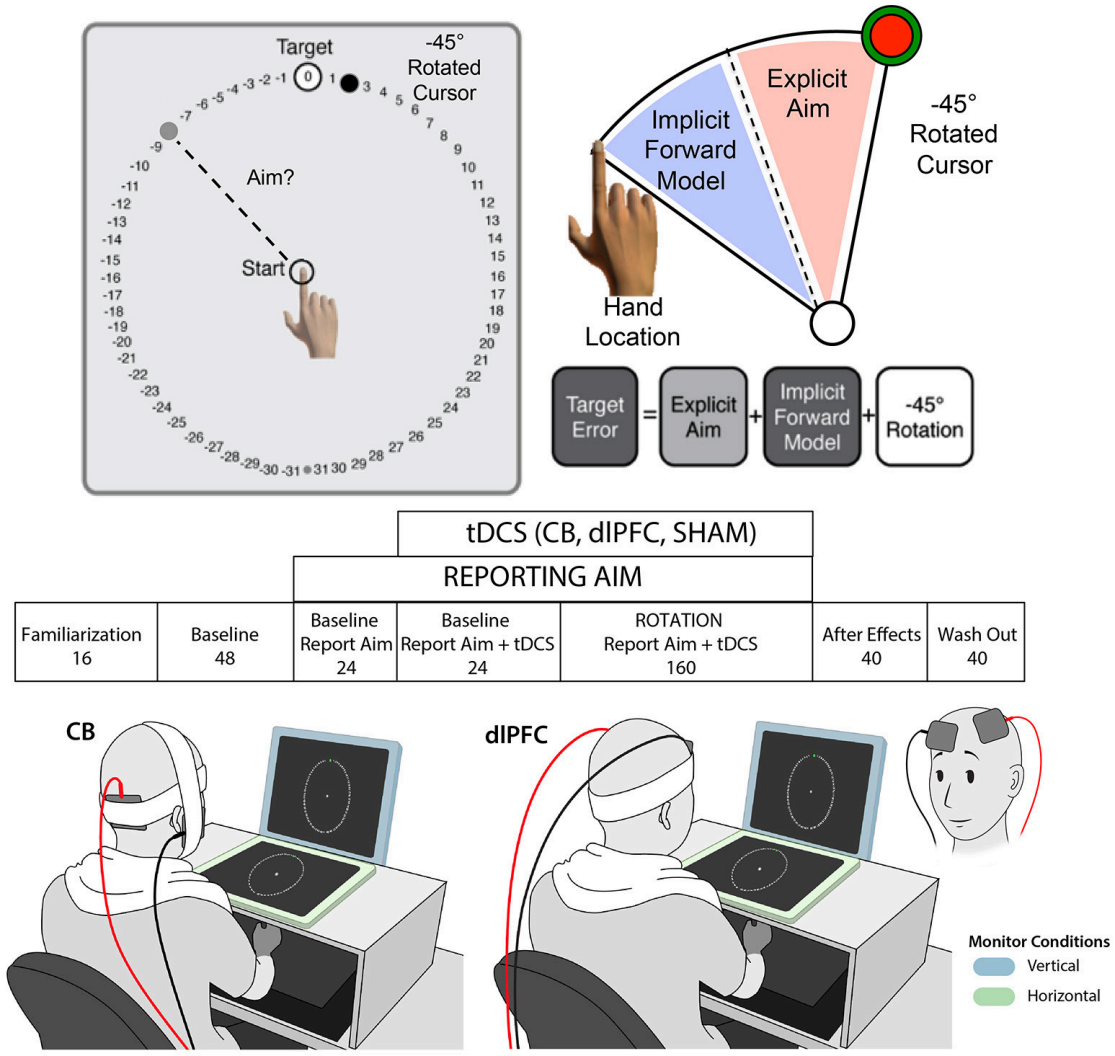
Experimental paradigm. Top: Visuomotor adaptation task with −45° clockwise rotation and numbers flanking target for reporting aim (adapted from Taylor et al., 2014). Middle: Experimental design with block name and number of trials listed below. Participants reported aim during blocks 3–5 and tDCS was turned on during blocks 4–5. Bottom: Examples of stimulation paradigm with vertical (blue) and horizontal (green) monitor contexts, and with the right cerebellar tDCS (left) and left dlPFC tDCS (right) electrode montages, are shown. Note that the dlPFC montage is also shown from the front, inset on the bottom far right, for greater clarity of the electrode positioning.

We used a paradigm adapted from Taylor et al. (2014), in which participants made center-out reaching movements with a digitizing stylus across a digitizing tablet (Wacom Intuos4 Extra Large) toward one of eight pseudo-randomized targets spaced equally around a circle, flanked by numbers, presented on an upright computer screen (see Figure 1). Participants were asked to try to get their visually-presented cursor (3.5 mm) to cross through the target, and to verbally report the number they were aiming at in order to get their cursor to the target before moving (e.g., during the rotation portion of the task, aiming toward −8 should allow them to land their cursor on the target). We measured the self-reported aiming as the explicit component, and from that, calculated the implicit component as the difference between the hand position and aiming angle (e.g., aiming toward−8, but actually moving the hand toward−6 would indicate a difference of 2, which was considered the implicit component). Importantly, participants were unable to see their hand during the task. Movement trajectories were sampled at 100 Hz, and stimuli were presented on a 1,280 × 1,024 pixel resolution LCD computer monitor (Dell), mounted 25 cm above the tablet.

### Reaching task

Each trial consisted of the participant localizing the stylus at the center of the tablet, visually guided by a white ring that reduced its dimensions as the stylus moved closer to the center. Once within 1 cm of a 5 mm starting circle, they were able to see the cursor and hone in on the starting circle. After holding the cursor in the starting circle for 1 s, a circle of 63 numbers (−31 to 31, each spaced 5.625° apart), 7 cm in radius appeared, with a green target circle (7 mm) at location 0. The green target circle appeared at one of eight locations, each spaced 45° apart (0, 45, 90, 135, 180, −135, −90, −45 degrees) and pseudorandomized so that each of the eight locations was presented in a random order every eight trials. Participants were asked to make movements as quickly and accurately as possible, and to make “slicing” movements through the target, to promote fast reaching movements. Participants were given feedback of their cursor (endpoint in Experiment 1; online in Experiments 2 and 3). With endpoint feedback, the cursor disappeared upon initial movement and only reappeared when the cursor crossed the circle, where it turned red and remained at its end location where it intersected the circle. With online feedback, the cursor remained visually present throughout the entire movement, turning red and remaining at its end location where it intersected the circle. This visual feedback, along with auditory feedback (a “ding” for successful trials, a buzzer for unsuccessful ones), provided information regarding task success. In addition, if the movement time from the initial movement to crossing the circle took longer than 400 ms, the participant heard an audio clip that said “Too slow!” to encourage them to move faster.

Participants completed seven blocks (see Figure 1). The first block was familiarization (Block 1; 16 trials) where they became acquainted with the reaching task. The next three blocks were baseline trials, with no rotation. They first completed a normal baseline block (Block 2; 48 trials), where they simply reached toward the target. Then they completed a baseline block while reporting their aim (Block 3; 24 trials), in which they said their aim aloud prior to each movement onset. Finally, they completed a baseline block with report and stimulation/sham (Block 4; 24 trials), to assess whether the stimulation had any effects on target error or aiming prior to the rotation. Starting at the beginning of block 4, the stimulation was turned on (CB, dlPFC, or SHAM; see below). Of note, to ensure that the experiment was double-blinded, a second study member came into the room to turn on/off the stimulation at the appropriate times. The stimulation remained on during the baseline with stimulation block (Block 4; 24 trials) and the rotation block (Block 5; 160 trials), during which a 45 degree clockwise rotation from actual hand position was introduced. Participants were not given any new instructions or informed of the correct direction to offset the rotation; they were only asked to continue trying to get their cursor on the target. At the conclusion of the rotation block, the second experimenter turned the stimulation off. During the last two blocks (Blocks 6 and 7), there was no reporting of aim, rotation of cursor, or stimulation. In Block 6 (40 trials), participants completed a block of no-feedback trials, during which they were asked simply to aim toward a green target that appeared without numbers flanking it and received no feedback about their performance except a “knocking” sound to let them know they crossed the circle. Finally, they completed a wash out block (Block 7; 40 trials), during which they again saw only a green circle but now received visual and audio feedback (“ding”/“buzz”) regarding their performance.

### Transcranial direct current stimulation

tDCS was delivered via a Chattanooga Ionto iontophoresis unit (EJO International, Surrey, UK) using two 5 × 5 cm sponge electrodes soaked with saline. 2 mA of anodal stimulation was applied during the baseline with stimulation block and rotation block (Blocks 4-5, 184 trials total), which lasted less than 25 min. Cerebellar (CB) stimulation utilized a bipolar electrode montage with the anode placed over the right cerebellar cortex, located 3 cm to the right of the inion, and the reference electrode placed over the right buccinator muscle (Galea et al., 2009). Dorsolateral prefrontal cortex (dlPFC) stimulation utilized a canonical bipolar electrode montage with the anode placement corresponding to the F3 position in the international 10–20 EEG system and reference placement over the right contralateral supraorbital region (Fregni et al., 2005; Nitsche et al., 2008). For the SHAM electrode placements, half of the individuals received the CB montage, and half received the dlPFC montage (randomized across the SHAM group). To induce the sensation of stimulation in the SHAM group, the stimulator was turned on for 30 s and current allowed to ramp up, then silently turned off with a 5 s fade out. As mentioned previously, the tDCS application was double-blinded, with a second experimenter coming in to turn on/off the tDCS at the appropriate times. The order of stimulation (CB, dlPFC, and SHAM) was randomized so that both the experimenter conducting the study and the participant were unaware if the participant received real or sham stimulation.

### Movement analysis

We assessed kinematics and performed all movement analyses with MATLAB (MATLAB R2013b, The MathWorks Inc., Natick, MA). To assess task performance, we analyzed the endpoint angle of the hand, and all movement trajectories, regardless of the actual target location, were rotated to a common reference axis with the target location set at 0°. The hand angle was computed by drawing a straight line between reference points positioned at 1 and 7 cm along the trajectory and computing the angle of this line. Positive angles indicate a counterclockwise deviation from the target and negative angles indicate a clockwise deviation from the target. These angles are reported in hand space. To characterize the process of learning, we averaged the hand angle during the rotation block (Block 5), normalized to the average of the last eight trials in the last baseline block to account for any hand bias (Block 4). To determine the size of the aftereffect for each group, we averaged the hand angle for the first eight trials in the no-feedback block (Block 6) and subtracted the average of the last eight trials in the last baseline block (Block 4) for each participant. Note that because the target locations were chosen in a pseudorandom fashion, the average of each epoch includes one reach to each of the eight target locations. In addition, since the sequence of target locations was randomized across participants, the averaging procedure removes any variability associated with specific target locations. We also measured reaction time, defined as the time between target onset and when the participant's hand position was 1 cm from the starting circle, and movement time, defined as the time required to traverse from the 1 cm position to the 7 cm position.

### Experiment 1

The goal of Experiment 1 was to elucidate the neural contributions of implicit and explicit learning during visuomotor adaptation, as measured by a task paradigm by Taylor et al. (2014). Participants were randomly assigned to three groups (CB (anodal stimulation over cerebellum), dlPFC (anodal stimulation over dorsolateral prefrontal cortex), or sham (SHAM); see *Transcranial direct current stimulation* for more details on stimulation parameters). We replicated the exact task paradigm from Taylor et al. (2014), using a horizontally presented stimulus screen, centered and fixed above a digitizing tablet, with endpoint feedback and instructions to report aim. Results from this experiment revealed an effect of dlPFC stimulation on improving visuomotor adaptation, with a weak increase on the explicit component and no effects on the implicit component. There were no significant effects of CB stimulation, which motivated the design of Experiment 2.

### Experiment 2

While Experiment 1 did not show significant effects of CB stimulation, previous work has shown that anodal stimulation over the cerebellum may significantly enhance visuomotor adaptation (e.g., Galea et al., 2011). The lack of cerebellar effects in Experiment 1 was concerning, although in line with more recent literature suggesting inconsistent effects of cerebellar stimulation on visuomotor adaptation (Jalali et al., 2017). We carefully studied the differences between the current and previous studies, which included differences in the total number of trials (fewer trials in the current study compared to Galea et al., 2011), the rotation angle (45° in the current study compared to 30° in Galea et al., 2011), type of feedback (endpoint in the current study vs. online in Galea et al., 2011), and screen orientation (horizontal in the current study vs. vertical in Galea et al., 2011). Although the number of trials could have influenced the effects, there were clear effects early within the rotation block in Galea et al. (2011), suggesting this was not a major factor. Regarding the rotation angle, numerous adaptation studies have used varying rotation angles or even different rotation paradigms (e.g., prism glasses, force field adaptations) and have shown cerebellar effects, suggesting that it should not be strongly affected by this factor either (e.g., Bernard and Seidler, 2013; Herzfeld et al., 2014). Therefore, there remained two key differences between this study and the original (Galea et al., 2011) study stood out as potential culprits for the lack of cerebellar effects: (1) the type of feedback (endpoint vs. online), and (2) the degree of visuomotor transformation required from the hand position to the feedback screen orientation (e.g., ours used a horizontal screen, which requires no transformation from hand position to feedback screen orientation; previous CB tDCS studies used a vertical screen, which require a 90° transformation from hand position to feedback screen orientation). The first issue was addressed in previous work by Taylor et al. (2014) showing that endpoint feedback biases individuals toward using more explicit learning while online feedback biases individuals toward using implicit learning. The second issue was addressed in the more recent (Jalali et al., 2017), in which they tested effects of CB tDCS vs. sham during visuomotor adaptation using either a vertical or horizontal screen. Overall, they found a stronger effect of CB tDCS compared to sham during the vertical, but not horizontal, condition; however, this was difficult to replicate and showed relatively modest effect sizes. Thus, in Experiment 2, we asked whether changing these two elements to replicate the previous CB tDCS study conditions (e.g., using a vertical screen and online feedback, as in Galea et al., 2011) would now bias individuals toward using greater implicit learning, and whether this in turn would enhance the effects of CB stimulation while downplaying the effects of dlPFC stimulation. We found some evidence to support the argument that CB stimulation improves visuomotor adaptation, primarily through implicit learning, although these effects were weak and not significant. However, now we also found no significant effects of dlPFC stimulation. This dissociation motivated the design of Experiment 3.

### Experiment 3

Based on results from Experiments 1 and 2, we then wanted to directly examine the effects of amount of visuomotor transformation (as indicated by screen orientation compared to hand position) on explicit and implicit mechanisms during visuomotor adaptation, while holding the type of feedback constant between groups (e.g., online feedback). Using the same task paradigm as in the first two experiments, we completed the same experiment with online feedback using a horizontal screen vs. a vertical screen. In all cases, the screen was positioned at midline and centered to match the center of the tablet. The task and procedures were identical between all groups. We had six groups (CONTEXT: Horizontal × Vertical, and STIM: CB × dlPFC × Sham), initially with *n* = 10 in each (HOR-CB, HOR-dlPFC, HOR-SHAM, VER-CB, VER-dlPFC, VER-SHAM). After outlier detection, which showed 1 outlier in each of the SHAM groups, we had *n* = 10 in all groups except the SHAM groups, which had *n* = 9. Data for the three vertical online groups were taken from Experiment 2, and an additional three groups were recruited to perform the experiment in the horizontal online condition. Based on previous results, we hypothesized that the horizontal groups would utilize a greater explicit component, due to ease of generating an explicit strategy in the horizontal condition. We expected the vertical groups to utilize a greater implicit component, due to the difficulty in explicitly generating a strategy for the more complex transformation. Our results support this hypothesis and suggest a strong context-dependent bias for overall visuomotor adaptation.

### Statistical analysis

Statistical analyses were carried out in SPSS (IBM SPSS Statistics, Version 25.0, IBM Corp., Armonk, NY) and using custom scripts in MATLAB (MATLAB R2013b, The Mathworks Inc., Natick, MA). We first assessed for outliers in each experiment, which were defined as any scores (target error, aiming angle, or, internal model) that fell beyond the mean plus or minus 3 times the standard deviation of the group. This yielded two outliers in Experiment 1 (1 in the PFC group, 1 in the SHAM group), one outlier in Experiment 2 (1 in the SHAM group), and one new outlier in Experiment 3 (1 in the new horizontal online SHAM group, along with the 1 outlier in the Experiment 2 vertical online SHAM group). For Experiments 1 and 2, we then assessed the effects of stimulation on motor performance using one-way ANOVAs across all stimulation groups (CB, dlPFC, SHAM) to separately examine differences in target error and reaction time for each context. These were performed during the baseline tDCS block (Block 4), to ensure no initial differences between groups, and during the rotation block (Block 5; relative to the last epoch of Block 4) to examine the effects of stimulation on adaptation. To explore whether these effects persisted after the rotation period, we also analyzed the first epoch of the no feedback block (aftereffects; Block 6; relative to the last epoch of baseline Block 4). *Post-hoc* Tukey tests were applied following significant main effects. In addition, since we anticipated that the main differences should occur between the stimulation groups compared to sham, in the event of non-significant ANOVAs, we also performed exploratory analyses by directly comparing between dlPFC/SHAM and CB/SHAM using independent samples *t*-tests. In Experiment 3, to analyze the interaction between the effects of stimulation and different contexts, we first analyzed differences between the two sham groups, in the absence of prolonged stimulation, using independent samples *t*-tests. We then performed a 2 × 3 ANOVA with between-group factors CONTEXT (HOR, VER) and STIM (CB, dlPFC, SHAM). *Post-hoc* tests were performed on pairwise comparisons when ANOVAs showed a significant effect. For all dependent measures, we report the mean and standard deviation for all dependent variables subjected to statistical evaluation. For all statistical analyses, we also report the effect size, which for ANOVAs is provided as partial eta squared (η_*p*_^2^) and Cohen's f (f), and for *t*-tests, is provided as both Cohen's d (d), a standard measure of effect size, and as Hedges' *g* (g), an unbiased measure of effect size that is more sensitive with smaller sample sizes. Notably, both Cohen's d and Hedges' g produced similar results. Results can also be found in Tables 1, 2. Finally, due to the small sample sizes and high variability, especially in the first two experiments, in addition to traditional null hypothesis testing, we also used a Bayesian analysis to explore the probability of the data given the alternative hypothesis vs. the probability of the data given the null hypothesis. Using the R package BayesFactor (Morey et al., 2015), we report the Bayes Factor (BF) for the primary analyses in Experiments 1 and 2. Briefly, the Bayes Factor provides a ratio of the strength of the present evidence in favor of the experimental effect vs. the null effect. The BF has been used in recent tDCS studies to provide evidence of the null hypothesis given the current data (e.g., Apšvalka et al., 2018). As it is a ratio, a BF of 10 suggests that, given the current data, the experimental effect is ten times more likely to occur than the null effect. Previous work has suggested that a BF of 0–3 should be considered weak, anecdotal evidence, a BF of 3–10 should be considered substantial evidence with a likely effect, and a BF of 10–30 should be considered strong evidence (Jeffreys, 1961).

**Table 1:** Results from Experiment 1 (left) and Experiment (2) right.

**Experiment 1: Endpoint feedback, horizontal screen**					**Experiment 2: Online feedback, vertical screen**				
**TARGET ERROR**					**TARGET ERROR**				
One-way ANOVA	*F*_(2, 43)_ = 3.40	*P* = 0.042^*^	η*_*p*_*^2^ = 0.14	f = 0.40	One-way ANOVA	*F*_(2, 26)_ = 1.55	*P* = 0.23	η*_*p*_*^2^ = 0.11	f = 0.35
	***n***	**mean**	**sd**			***n***	**mean**	**sd**	
CB	16	−1.00	4.11		CB	10	−2.67	4.20	
PFC	15	1.41	2.51		PFC	10	−4.79	5.01	
SHAM	15	−3.94	8.53		SHAM	9	−6.61	5.44	
***post-hoc*** **Tukey Tests**					**A priori** ***t*****-tests**				
	***p-value***	**Cohen's d**	**Hedges' g**			***t*****-statistic**	***p-value***	**Cohen's d**	**Hedges' g**
CB x PFC	0.46	0.71	0.68		CB x SHAM	*t*_(17)_ = 1.78	0.09	0.81	0.78
CB x SHAM	0.32	0.44	0.43		PFC x SHAM	*t*_(17)_ = 0.76	0.46	0.35	0.33
PFC x SHAM	0.03^*^	0.85	0.83						
**EXPLICIT AIMING ANGLE**					**EXPLICIT AIMING ANGLE**				
**One-way ANOVA**	*F*_(2, 43)_ = 2.03	*p* = 0.14	η*p*^2^ = 0.086	f = 0.31	**One-way ANOVA**	*F*_(2, 26)_ = 1.33	*p* = 0.28	η*p*^2^ = 0.09	f = 0.31
	***n***	**mean**	**sd**			***n***	**mean**	**sd**	
CB	16	29.42	5.78		CB	10	10.15	11.36	
PFC	15	33.35	6.37		PFC	10	17.26	6.33	
SHAM	15	28.73	8.09		SHAM	9	15.56	11.99	
**A priori** ***t*****-tests**					**A priori** ***t*****-tests**				
	***t*****-statistic**	***p-value***	**Cohen's d**	**Hedges' g**		***t-*****statistic**	***p-value***	**Cohen's d**	**Hedges' g**
CB × SHAM	*t*_(29)_ = 0.27	0.79	0.10	0.10	CB × SHAM	*t*_(17)_ = −1.01	0.33	0.46	0.44
PFC × SHAM	*t*_(28)_ = 1.74	0.09	0.63	0.62	PFC × SHAM	*t*_(17)_ = 0.39	0.70	0.18	0.17
**SUBTRACTION OF AIMING ANGLE FROM TARGET ERROR (IMPLICIT)**					**SUBTRACTION OF AIMING ANGLE FROM TARGET ERROR (IMPLICIT)**				
One-way ANOVA	*F*_(2, 43)_ = 0.82	*p* = 0.45	η*p*^2^ = 0.037	f = 0.20	One-way ANOVA	*F*_(2, 26)_ = 2.93	*p* = 0.071	η*p*^2^ = 0.18	f = 0.47
	***n***	**mean**	**sd**			***n***	**mean**	**sd**	
CB	16	15.29	5.48		CB	10	31.25	8.40	
PFC	15	12.79	5.86		PFC	10	22.84	6.84	
SHAM	15	13.41	5.74		SHAM	9	23.10	10.84	
**A priori** ***t*****-tests**					**A priori** ***t*****-tests**				
	***t-*****statistic**	***p-value***	**Cohen's d**	**Hedges' g**		***t-*****statistic**	***p-value***	**Cohen's d**	**Hedges' g**
CB × SHAM	*t*_(29)_ = 0.94	0.36	0.34	0.33	CB × SHAM	*t*_(17)_ = 1.84	0.083	0.84	0.81
PFC × SHAM	*t*_(28)_ = −0.29	0.77	0.11	0.10	PFC × SHAM	*t*_(17)_ = 1.74	0.95	0.03	0.03

*Values for target error, explicit aiming angle, and subtraction of aiming angle (implicit component), all in units of degrees, during the rotation block, provided with mean and standard deviations (sd) for each experiment and group (CB, cerebellum; PFC, dorsolateral prefrontal cortex; SHAM, sham control group). Significant p-values of p < 0.05 are shown in red; p-values of p < 0.10 are shown in orange*.

**Table 2:** Results from Experiment 3A (SHAM groups only) and Experiment 3B (All groups) right.

**Experiment 3A: Vertical vs. horizontal screen**				**Experiment 3B: Context x stim groups (CB, PFC, SHAM)**				
**TARGET ERROR**				**TARGET ERROR**				
***T*****-test**	*t*_(16)_ = 2.66, *p* = 0.017^*^	Cohen's d = 1.26	Hedges' g = 1.20	2 × 3 ANOVA (context x STIM)	*F*_(2, 52)_ = 1.16	*p* = 0.32	η*p*^2^ = 0.04	f = 0.20
	***n***	**mean**	**sd**	Main effect of context	*F*_(2, 52)_ = 8.34	p = 0.006^*^	η*p*^2^ = 0.14	f = 0.40
Vertical Sham	9	−6.61	5.44	Main effect of STIM	*F*_(2, 52)_ = 1.48	*p* = 0.24	η*p*^2^ = 0.05	f = 0.23
Horizontal Sham	9	−1.45	2.03		**Vertical Screen**		**Horizontal Screen**	
					**mean**	**sd**	**mean**	**sd**
				CB (*n* = 10)	−2.67	4.20	−1.28	3.31
				PFC (*n* = 10)	−4.79	5.01	−2.40	2.29
				SHAM (*n* = 9)	−6.61	5.44	−1.45	2.03
**EXPLICIT AIMING ANGLE**				**EXPLICIT AIMING ANGLE**				
***T*****-test**	t_(16)_ = 1.50, *p* = 0.15	cohen's d = 0.71	hedges' g = 0.68	2 × 3 ANOVA (context × STIM)	F_(2, 52)_ = 1.51	*p* = 0.23	ηp^2^ = 0.06	f = 0.25
	***n***	**mean**	**sd**	Main effect of context	F_(2, 52)_ = 17.62	*p* < 0.001^*^	η*p*^2^ = 0.25	f = 0.58
Vertical sham	9	22.48	6.81	Main effect of STIM	F_(2, 52)_ = 0.49	*p* = 0.62	η*p*^2^ = 0.02	f = 0.14
Horizontal sham	9	15.56	11.99		**Vertical Screen**		**Horizontal Screen**	
					**mean**	**sd**	**mean**	**Sd**
				CB (*n* = 10)	10.15	11.36	26.55	8.65
				PFC (*n* = 10)	17.26	6.33	25.10	9.91
				SHAM (*n* = 9)	15.56	11.99	22.48	6.81
**SUBTRACTION OF AIMING ANGLE FROM TARGET ERROR (IMPLICIT)**				**SUBTRACTION OF AIMING ANGLE FROM TARGET ERROR (IMPLICIT)**				
***T*****-test**	*t*_(16)_ = −0.50, *p* = 0.62	Cohen's d = 0.24	Hedges' g = 0.23	2 × 3 ANOVA (context x STIM)	*F*_(2, 52)_ = 2.68	*p* = 0.08	η*p*^2^ = 0.09	f = 0.31
	***n***	**mean**	**sd**	Main effect of context	*F*_(2, 52)_ = 11.09	*p* = 0.002^*^	η*p*^2^ = 0.18	f = 0.47
Vertical sham	9	20.98	6.46	Main effect of STIM	*F*_(2, 52)_ = 1.21	*p* = 0.31	η*p*^2^ = 0.05	f = 0.23
Horizontal sham	9	23.10	10.84		**Vertical screen**		**Horizontal screen**	
					**mean**	**sd**	**mean**	**Sd**
				CB (*n* = 10)	31.25	8.40	17.05	7.13
				PFC (*n* = 10)	22.84	6.84	17.26	9.56
				SHAM (*n* = 9)	23.10	10.84	20.98	6.46

*Values for target error, explicit aiming angle, and subtraction of aiming angle (implicit component), all in units of degrees, during the rotation block, provided with mean and standard deviations (sd) for each experiment and group (CB, cerebellum; PFC, dorsolateral prefrontal cortex; SHAM, sham control group). Significant p-values of p < 0.05 are shown in red; p-values of p < 0.10 are shown in orange*.

To examine the effects of stimulation on explicit (aiming angle) and implicit (hand angle minus aiming angle) learning, we used a one-way ANOVA with GROUP (CB, dlPFC, SHAM) on each mechanism separately. Again, we hypothesized that the stimulation conditions would weight the two real stimulation groups (CB, dlPFC) toward one mechanism or the other, while the SHAM group should not change. Therefore, we also performed exploratory analyses of analyzing the direct comparison between CB/dlPFC groups against SHAM, using independent samples *t*-tests, to understand if there was any stimulation-induced difference compared to a baseline control group.

## Results

### Experiment 1

#### dLPFC stimulation reduces target error compared to sham

**Figure 2:**
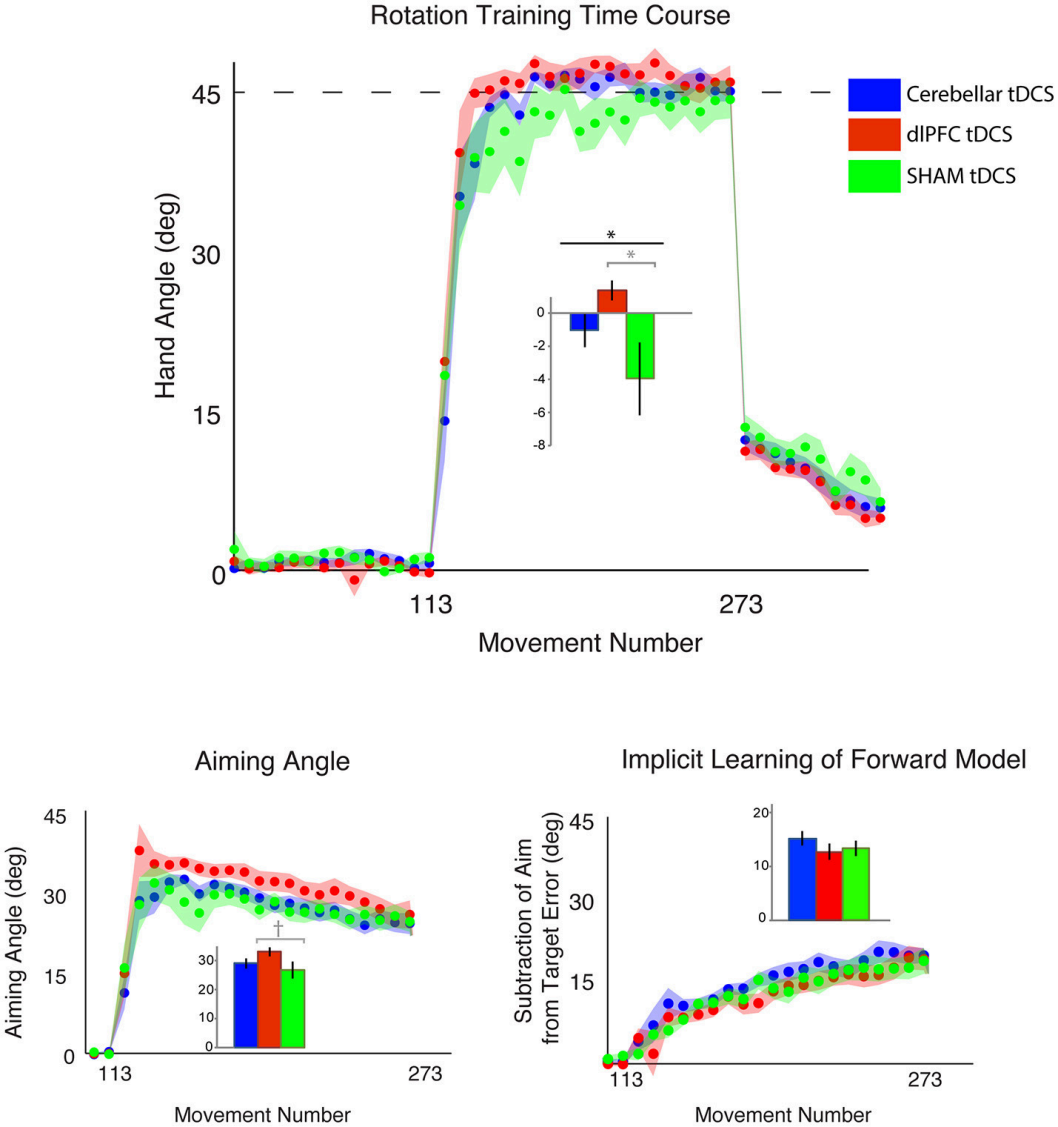
Results from Experiment 1, with a horizontal screen context and endpoint feedback. Top: Hand angle plotted across groups (CB = blue, dlPFC = red, SHAM = green) across all trials. Dotted line indicates 45° rotation required to minimize target error to zero. Bottom left: Explicit learning as indicated by aiming angle across groups during the rotation block (measured as participant self-reported aim per trial) plotted across all groups. Bottom right: Implicit learning across groups during the rotation block and after-effects/wash out period, measured as the subtraction of the aiming angle from target error. Data points represent epochs of eight movements; shading represents the standard error of the mean for each epoch. Bar graphs represent average per group across rotation block, with degrees on the y-axis, standard error of the mean shown in error bars, ANOVA results indicated in black, *t*-test results indicated in gray. Significance of *p* < 0.05 indicated as^*^, trends toward significance (*p* < 0.10) indicated as†.

We first compared the effects of dlPFC, CB, and SHAM stimulation on target error during the visuomotor adaptation task. In line with our hypotheses, there was a significant difference in target error between groups during the rotation block [one-way ANOVA: *F*_(2, 43)_ = 3.40, *p* = 0.042, η_*p*_^2^ = 0.14, f = 0.40, BF = 1.65 ± 0.02%; CB = −1.00 ± 4.11°, dlPFC = 1.41 ± 2.51°, SHAM = −3.94 ± 8.53°; Figure 2; Table 1]. *Post-hoc* Tukey tests revealed that the dlPFC group had a significantly reduced target error compared to SHAM (*p* = 0.033, d = 0.85, g = 0.83). There were no significant differences between dlPFC and CB groups (*p* = 0.46, d = 0.71, g = 0.68), nor between CB and SHAM groups (*p* = 0.32, d = 0.44, g = 0.43). Importantly, the groups were not significantly different in terms of target error during the baseline block with stimulation [Block 4; one-way ANOVA: *F*_(2, 43)_ = 1.60, *p* = 0.21, η_*p*_^2^ = 0.07, f = 0.27; CB = 0.40 ± 1.13°, dlPFC = −0.17 ± 1.06°, Sham = 0.62 ± 1.54°] or in terms of aftereffects immediately following the rotation [one-way ANOVA: *F*_(2, 43)_ = 0.15, *p* = 0.86, η_*p*_^2^ = 0.007, f = 0.08; CB = 11.67 ± 4.79°, dlPFC = 11.49 ± 3.03°, SHAM = 12.29 ± 4.46°]. There were also no differences in reaction time across groups during baseline with stimulation [F_(2, 43)_ = 1.49, *p* = 0.24, CB = 1.17 ± 0.38 s, dlPFC = 1.51 ± 0.76 s, SHAM = 1.33 ± 0.47 s], rotation [*F*_(2, 43)_ = 1.63, *p* = 0.21; CB = 1.74 ± 0.53 s, dlPFC = 2.08 ± 0.58 s, SHAM = 1.94 ± 0.47 s], or aftereffect [*F*_(2, 43)_ = 0.76, *p* = 0.47, CB = 0.66 ± 0.30 s, dlPFC = 0.57 ± 0.16 s, SHAM = 0.68 ± 0.27 s] blocks.

### No significant effects specifically on explicit or implicit learning

We then examined the effects of CB, dlPFC, and SHAM stimulation on implicit and explicit mechanisms. We found only a trend but no significant effects of explicit aiming between groups [one-way ANOVA *F*_(2, 43)_ = 2.03, *p* = 0.14, η_*p*_^2^ = 0.086, f = 0.31, BF = 0.66 ± 0.03%; CB = 29.42 ± 5.78°, dlPFC = 33.35 ± 6.37°, SHAM = 28.73 ± 8.09°; Figure 2]. We then performed a prior *t*-tests between stimulation groups and SHAM directly. We found a moderate increase in explicit learning in the dlPFC group compared to SHAM, but this difference was not significant [*t*_(28)_ = 1.74, *p* = 0.09, d = 0.63, g = 0.62]. There were no differences between CB and SHAM groups [*t*_(29)_ = 0.27, *p* = 0.79, d = 0.10, g = 0.10].

The implicit component also did not show any significant differences between the groups [*F*_(2, 43)_ = 0.82, *p* = 0.45, η_*p*_^2^ = 0.037, f = 0.20, BF = 0.29 ± 0.04%; CB = 15.29 ± 5.48°, dlPFC = 12.79 ± 5.86°, SHAM = 13.41 ± 5.74°]. A priori *t*-tests did not reveal any differences between stimulation groups and sham: CB and SHAM [*t*_(29)_ = 0.94, *p* = 0.36, d = 0.34, g = 0.33], dlPFC and SHAM [*t*_(28)_ = −0.29, *p* = 0.77, d = 0.11, g = 0.10; Figure 2].

**Figure 3:**
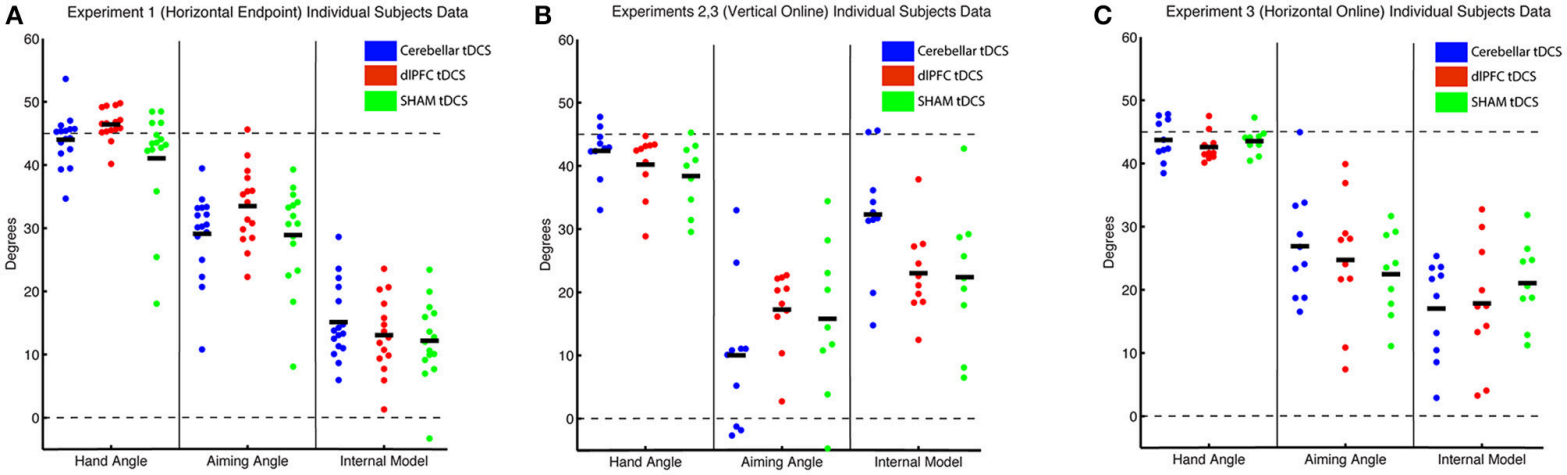
Individual subject data for each experiment, where each subject is represented as 1 circle, and group means are indicated by short solid black lines (CB = blue, dlPFC = red, SHAM = green). Data for hand angle, aiming angle, and internal model are shown for each experiment. **(A)** Individual subject data from Experiment 1 (horizontal screen, endpoint feedback). **(B)** Individual subject data from Experiment 2 (vertical screen, online feedback), which was also used in Experiment 3. **(C)** Individual subject data from Experiment 3 (horizontal screen, online feedback).

Thus, while we saw a significant difference in target error, primarily between dlPFC and Sham groups, the underlying mechanisms behind this difference could not be cleanly attributed to the explicit component, which was only slightly, and not significantly, increased in the dlPFC group vs. Sham. The low Bayes Factor also suggests that this effect across the groups is relatively weak. We then performed a closer examination of individual subject results for both target error and aiming, and found that both showed large interindividual variability and suggested that effects were driven by a few individuals in each group (large target error and low aiming in the SHAM group and low target error and high aiming in the dlPFC group; Figure 3A). However, the majority of the participants showed similar ranges of results. This suggests that the effects were driven primarily by a few poor performers in the SHAM group and a few strong performers in the dlPFC group, but not by consistent effects within each group.

Although these results were in line with more recent findings of inconsistent modulations on motor learning by tDCS (Jalali et al., 2017; Lopez-Alonso et al., 2018), we also wondered whether the lack of clear effects and complete lack of CB effects was due to contextual differences underlying our experiment compared to previous research showing significant CB effects (e.g., Galea et al., 2011).

### Experiment 2

Given the discrepant findings between Experiment 1 and previous literature (e.g., Galea et al., 2011; Jalali et al., 2017) regarding the mixed effects of CB stimulation on target error during visuomotor adaptation (discussed in *Methods: Experiment 2*), we performed a second experiment. Here, we replicated the same experimental paradigm as in Experiment 1, but with two changes to match the set-up of previous reports showing CB stimulation improvements on visuomotor adaptation. First, we used online feedback, instead of endpoint feedback, which has been shown to engage more implicit mechanisms (Taylor et al., 2014), and second, we used a vertical, rather than horizontal, feedback screen, which required an additional visuomotor transformation between hand space and visual feedback. We hypothesized that in doing so, we should bias participants to utilize more implicit learning and thus show greater effects from CB stimulation, if CB stimulation was indeed related to implicit learning.

### CB stimulation weakly reduces target error compared to sham

**Figure 4:**
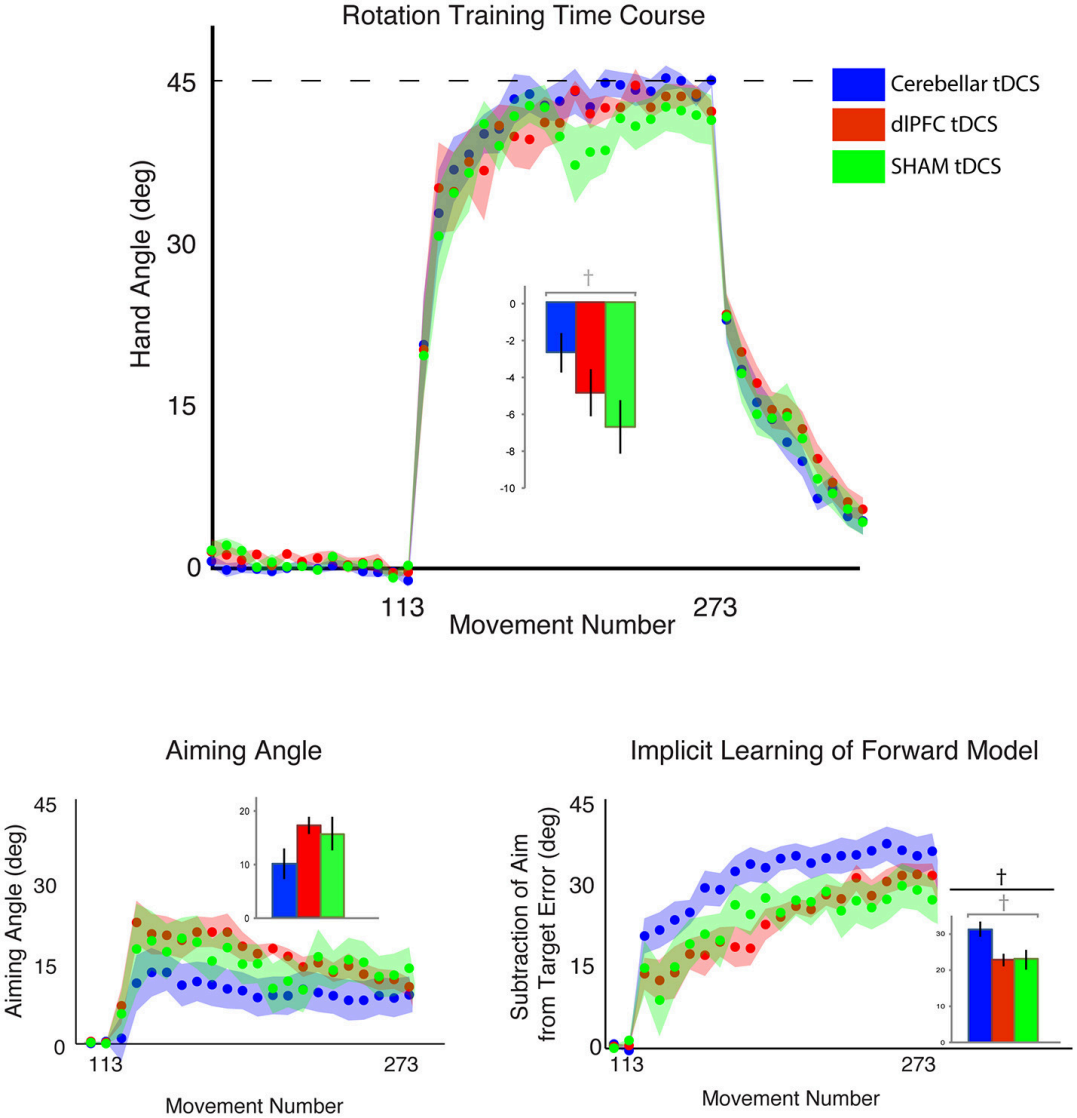
Results from Experiment 2, with a vertical screen context and online feedback. Top: Hand angle plotted across groups (CB = blue, dlPFC = red, SHAM = green) across all trials. Dotted line indicates 45° rotation required to minimize target error to zero. Bottom left: Explicit learning as indicated by aiming angle across groups during the rotation block (measured as participant self-reported aim per trial) plotted across all groups. Bottom right: Implicit learning across groups during the rotation block and after-effects/wash out period, measured as the subtraction of the aiming angle from target error. Data points represent epochs of eight movements; shading represents the standard error of the mean for each epoch. Bar graphs represent average per group across rotation block, with degrees on the y-axis, standard error of the mean shown in error bars, ANOVA results indicated in black, *t*-test results indicated in gray. Significance of *p* < 0.05 indicated as^*^, trends toward significance (*p* < 0.10) indicated as†.

The outlier detection yielded 1 outlier in the Sham group, resulting in *n* = 10 for CB and dlPFC groups and *n* = 9 for Sham. Under these new experimental conditions, we again examined target error across the three stimulation groups (CB, dlPFC, SHAM) but now did not find a significant difference in target error during the rotation block (one-way ANOVA: [*F*_(2, 26)_ = 1.55, *p* = 0.23, η_*p*_^2^ = 0.11, f = 0.35, BF = 0.57 ± 0.01%; CB = −2.67 ± 4.20°, dlPFC = −4.79 ± 5.01°, SHAM = −6.61 ± 5.44°; Figure 4). A priori *t*-tests showed that the CB group had a nonsignificant trend toward reducing target error compared to the SHAM group [*t*_(17)_ = 1.78, *p* = 0.093, d = 0.81, g = 0.78]. In addition, there was no significant difference in target error between dlPFC and SHAM groups [*t*_(17)_ = 0.76, *p* = 0.46, d = 0.35, g = 0.33]. As in Experiment 1, there were no significant differences across groups in target error at baseline [*F*_(2, 26)_ = 0.98, *p* = 0.39, η_*p*_^2^ = 0.07, f = 0.27], and no significant differences across groups in after-effects [*F*_(2, 26)_ = 0.07, *p* = 0.93, η_*p*_^2^ = 0.006, f = 0.08]. There was no significant difference in reaction time across groups at baseline [*F*_(2, 26)_ = 0.16, *p* = 0.86, CB = 0.87 ± 0.36 s, dlPFC = 0.81 ± 0.54 s, SHAM = 0.92 ± 0.32 s], during the rotation block [*F*_(2, 26)_ = 1.94, *p* = 0.16, CB = 1.15 ± 0.49 s, dlPFC = 1.19 ± 0.47 s, SHAM = 1.62 ± 0.74 s], or in the aftereffect [*F*_(2, 26)_ = 1.31, *p* = 0.29, CB = 0.45 ± 0.12 s, dlPFC = 0.48 ± 0.16 s, SHAM = 0.56 ± 0.18 s].

Due to these insignificant effects, we then ran a power analyses to examine, based on the target error effect size during adaptation between just CB and SHAM groups (d = 0.81), how many subjects would be needed to show a significant effect. Using an alpha = 0.05, and power = 0.80, to show a difference between CB and SHAM groups, *n* = 25 participants per group would be needed. This effect size is in line with recent studies showing similar effect sizes between anodal cerebellar tDCS and SHAM groups on visuomotor adaptation (e.g., Cohen's d = 0.7 in Jalali et al., 2017) or across different types of behaviors (e.g., meta-analysis showing Hedge's g = 0.59 in Oldrati and Schutter, 2017). Thus, altogether, we found only weak to null effects of CB stimulation on reducing target error compared to the SHAM group, similar to previous findings. However, we now also found no effects of dlPFC stimulation.

### CB stimulation weakly improves implicit, but not explicit, learning

We then examined stimulation effects on implicit and explicit components of motor learning. There was a group difference in the implicit contributions to learning the task, but this was not statistically significant [one-way ANOVA: *F*_(2, 26)_ = 2.93, *p* = 0.071, η_*p*_^2^ = 0.18, f = 0.47, BF = 1.31 ± 0.01%; CB = 31.25 ± 8.40°, dlPFC = 22.84 ± 6.84°; SHAM = 23.10 ± 10.84°]. A prior *t*-tests showed that the CB group relied more on implicit learning mechanisms compared to SHAM, but this also fell short of significance [*t*_(17)_ = 1.84, *p* = 0.083, d = 0.84, g = 0.81]. There were no significant differences in implicit learning between dlPFC and SHAM groups [*t*_(17)_ = 1.74, *p* = 0.95, d = 0.03, g = 0.03; Figure 4].

In addition, there were no significant group effects in explicit learning [one-way ANOVA: *F*_(2, 26)_ = 1.33, *p* = 0.28, η_*p*_^2^ = 0.09, f = 0.31, BF = 0.50 ± 0.01%; CB = 10.15 ± 11.36°, dlPFC = 17.26 ± 6.33°, SHAM = 15.56 ± 11.99°], and a priori *t*-tests revealed no differences between either CB and SHAM [t_(17)_ = −1.01, *p* = 0.33, d = 0.46, g = 0.44], or dlPFC and SHAM [*t*_(17)_ = 0.39, *p* = 0.70, d = 0.18, g = 0.17; Figure 4]. Thus, overall, in the vertical online context, we found only a weak effect of CB stimulation on facilitating implicit mechanisms of learning. In addition, opposite to Experiment 1, dlPFC stimulation now showed no effects on target error and had no effects on explicit mechanisms in this revised context. We also examined individual subject data and again found wide variability within groups, with effects that were primarily driven by a few individuals (Figure 3B).

### Experiment 3

The only changes between Experiments 1 and 2 were change in feedback type (from endpoint to online) and change in the amount of visuomotor transformation required between the hand space and visual feedback as modulated by screen orientation (horizontal to vertical). While previous research had shown that online feedback results in higher implicit learning and endpoint feedback results in higher explicit learning (Taylor et al., 2014), this difference could not explain the magnitude of changes observed. Thus, we hypothesized that the screen orientation also affected the relative contributions of implicit and explicit mechanisms used. Specifically, a vertical screen orientation might require a greater degree of visuomotor transformation from one's hand position to the feedback position (e.g., a 90° transformation), while a horizontal screen orientation provides a simpler transformation (e.g., a 1-to-1 match of one's actions with the visual feedback). Indeed, recent work has shown that CB tDCS has modest effects on visuomotor adaptation in a vertical screen condition, but not in a horizontal screen condition (Jalali et al., 2017). However, this study did not examine the effects of horizontal or vertical context on dlPFC stimulation. In Experiment 3 we directly examined the effects of the degree of visuomotor transformation on implicit/explicit mechanisms. Keeping feedback type constant (using online feedback for all groups), we added three new groups (Horizontal Online paradigm with CB/dlPFC/SHAM stimulation groups) and compared the findings to the previous three groups (Vertical Online paradigm with CB/dlPFC/SHAM stimulation from Experiment 2). In this way, we could examine CONTEXT (Horizontal/Vertical) by STIMULATION (CB/dlPFC/SHAM) interactions across the six groups. Based on our results from Experiments 1 and 2, we anticipated that the horizontal condition might increase explicit contributions, while the vertical condition might increase implicit contributions.

### Vertical context increases target error compared to horizontal context

**Figure 5:**
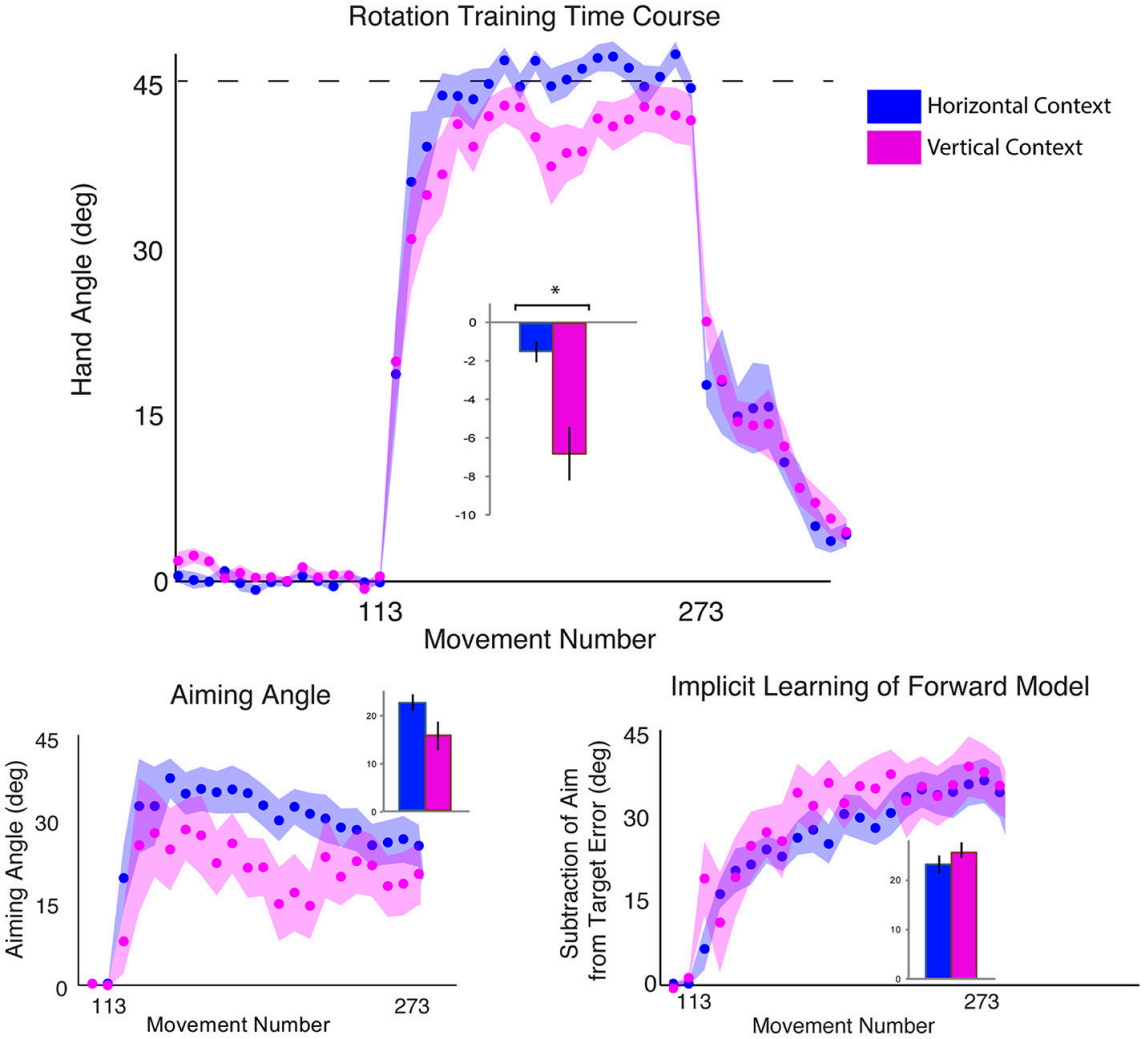
Results from Experiment 3, SHAM group only, comparing a horizontal screen context (blue) and a vertical screen context (magenta). Top: Hand angle plotted across groups (horizontal = blue, vertical = magenta) across all trials. Dotted line indicates 45° rotation required to minimize target error to zero. Bottom left: Explicit learning as indicated by aiming angle across groups during the rotation block (measured as participant self-reported aim per trial) plotted across all groups. Bottom right: Implicit learning across groups during the rotation block and after-effects/wash out period, measured as the subtraction of the aiming angle from target error. Data points represent epochs of eight movements; shading represents the standard error of the mean for each epoch. Bar graphs represent average per group across rotation block, with degrees on the y-axis, standard error of the mean shown in error bars, *t*-test results indicated in black. Significance of *p* < 0.05 indicated as^*^, trends toward significance (*p* < 0.10) indicated as†.

We first examined the effects of the vertical vs. horizontal context on visuomotor adaptation, without any effects of stimulation. To do this, we used the horizontal (HOR) and vertical (VER) SHAM groups, who received only 30 s of stimulation, which has previously been shown to induce the feeling of stimulation without actual longer-term facilitatory effects (Gandiga et al., 2006). We found that the participants performed significantly worse, as measured by greater target error during the rotation block, in the vertical condition compared to the horizontal condition [*t*_(16)_ = 2.66, *p* = 0.017, d = 1.26, g = 1.20; HOR = −1.45 ± 2.03°, VER = −6.61 ± 5.44°; Figure 4]. However, we did not find a significant difference in implicit learning between vertical and horizontal conditions for the sham groups [*t*_(16)_ = −0.50, *p* = 0.62, d = 0.24, g = 0.23], which showed very similar values (HOR = 20.98 ± 6.46°, VER = 23.10 ± 10.84°). There was also not a significant decrease in explicit learning in the vertical, compared to horizontal, conditions on average [t_(16)_ = 1.50, *p* = 0.15, d = 0.71, g = 0.68; HOR = 22.48 ± 6.81°, VER = 15.56 ± 11.99°; Figure 5]. As neither the implicit or explicit mechanisms showed significant differences, we then investigated single subject data, which showed interindividual high variance, especially in the vertical group (vertical sham data shown in Figure 3B in green, horizontal sham data shown in Figure 3C in green).

### Greater visuomotor transformation increases target error across stimulation groups

**Figure 6:**
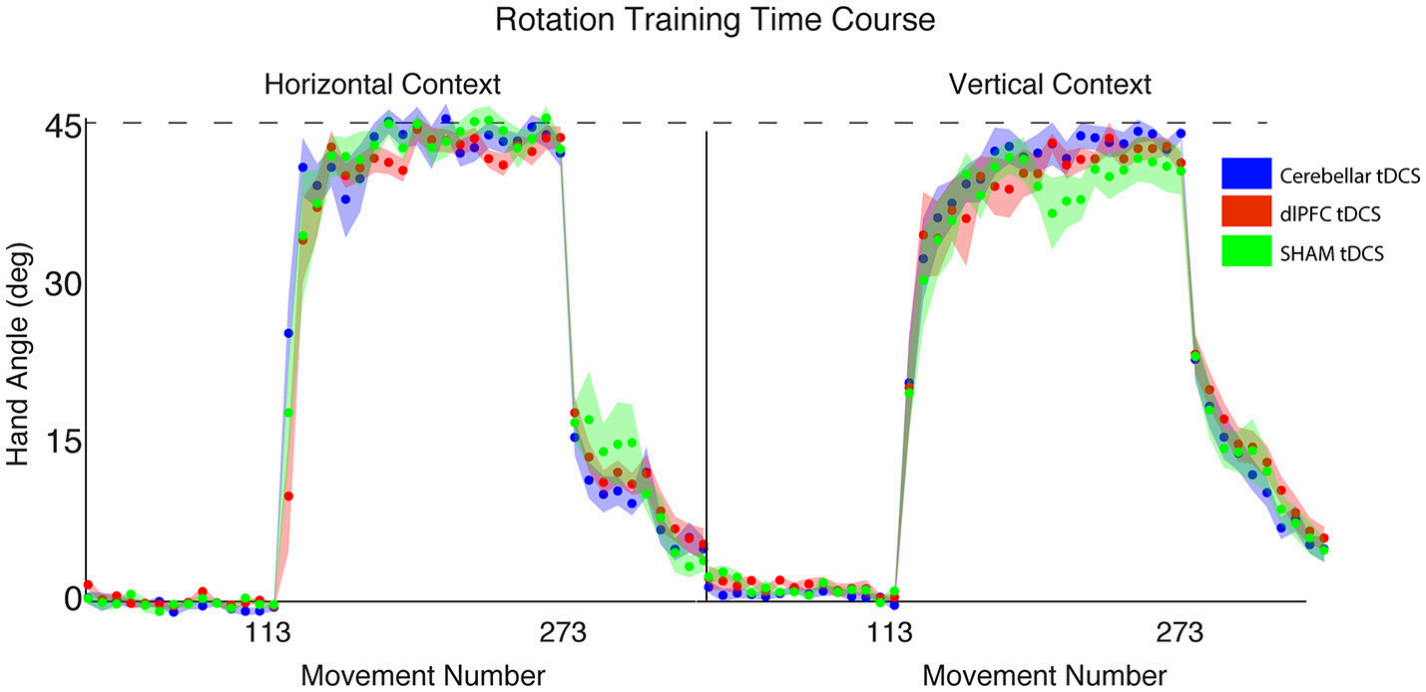
Results from Experiment 3, examining the effects of stimulation group by context. Hand angle plotted across stimulation groups (CB = blue, dlPFC = red, SHAM = green) across all trials. Dotted line indicates 45° rotation required to minimize target error to zero. Left: Hand angle during horizontal screen context. Right: Hand angle during vertical screen context. Data points represent epochs of eight movements; shading represents the standard error of the mean for each epoch.

We then examined the relationship between visuomotor transformation context (HOR, VER) and stimulation groups (CB, dlPFC, SHAM) in a 2 × 3 between-subjects ANOVA. There was no significant interaction between stimulation groups and screen context on target error [3 × 2 ANOVA with between-group factors CONTEXT and STIM: *F*_(2, 52)_ = 1.16, *p* = 0.32, η_*p*_^2^ = 0.04, f = 0.20]. There was, however, a significant main effect of CONTEXT [*F*_(2, 52)_ = 8.34, *p* = 0.006, η_*p*_^2^ = 0.14, f = 0.40; Figure 6], with all stimulation groups performing worse and showing greater target error in the vertical screen condition compared to the horizontal screen condition (HOR = −1.72 ± 2.56°, VER = −4.62 ± 4.98°). This suggests that the vertical condition is more difficult overall across groups, regardless of stimulation. There was no significant main effect of STIM [*F*_(2, 52)_ = 1.48, *p* = 0.24, η_*p*_^2^ = 0.05, f = 0.23]. The target error for each group is as follows: HOR: GROUP = −1.72 ± 2.56°, CB = −1.28 ± 3.31°, dlPFC = −2.40 ± 2.29°, SHAM = −1.45 ± 2.03°; VER: GROUP = −4.62 ± 4.98°, CB = −2.67 ± 4.20°, dlPFC = −4.79 ± 5.01°, SHAM = −6.61 ± 5.44°.

### Cerebellar stimulation increases implicit contributions, only in vertical context

**Figure 7:**
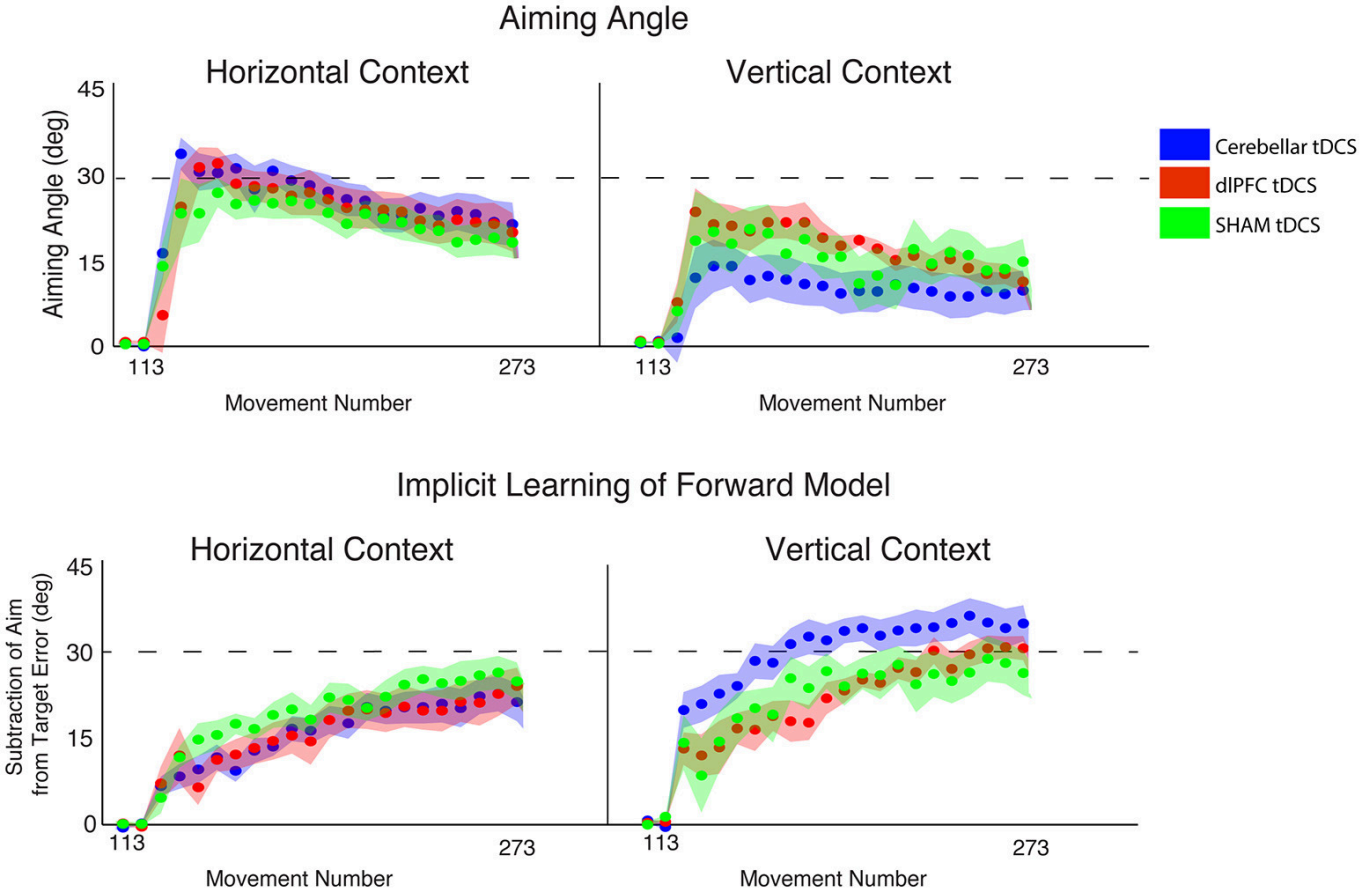
Results from Experiment 3, examining the effects of stimulation group by context. Top: Explicit learning as indicated by aiming angle across groups (CB = blue, dlPFC = red, SHAM = green) during the rotation block (measured as participant self-reported aim per trial) plotted across all groups (left = horizontal screen context, right = vertical screen context). Bottom: Implicit learning across groups during the rotation block and after-effects/wash out period, measured as the subtraction of the aiming angle from target error (left = horizontal screen context, right = vertical screen context). Data points represent epochs of eight movements; shading represents the standard error of the mean for each epoch.

Finally, we examined the effects of stimulation and context specifically on implicit and explicit mechanisms (2 × 3 ANOVAs, factors CONTEXT, STIM, for implicit and explicit learning, respectively). For implicit learning, we found an interaction between CONTEXT and STIM that fell short of significance [*F*_(2, 52)_ = 2.68, *p* = 0.078, η_*p*_^2^ = 0.09, f = 0.31] and a significant main effect of CONTEXT [*F*_(2, 52)_ = 11.09, *p* = 0.002, η_*p*_^2^ = 0.18, f = 0.47], but no significant main effect of STIM [*F*_(2, 52)_ = 1.21, *p* = 0.31, η_*p*_^2^ = 0.05, f = 0.23; Figure 7]. Specifically, implicit learning in the CB group was significantly increased compared to dlPFC and SHAM groups in the vertical, but not horizontal, context (HOR: GROUP = 18.34 ± 7.80°, CB = 17.05 ± 7.13°, dlPFC = 17.26 ± 9.56°, SHAM = 20.98 ± 6.46°; VER: GROUP = 25.81 ± 9.35°, CB = 31.25 ± 8.40°, dlPFC = 22.84 ± 6.84°, SHAM = 23.10 ± 10.84°). In other words, and in line with our previous experiments, this analysis showed that the effects of CB stimulation on increasing implicit learning were only weakly evident in the vertical context, but not the horizontal context. In contrast, for explicit learning, we found only a significant main effect of CONTEXT [*F*_(2, 52)_ = 17.62, *p* < 0.001, η_*p*_^2^ = 0.25, f = 0.58] and no interaction [*F*_(2, 52)_ = 1.51, *p* = 0.23, η_*p*_^2^ = 0.06, f = 0.25] or main effect of STIM [*F*_(2, 52)_ = 0.49, *p* = 0.62, η_*p*_^2^ = 0.02, f = 0.14; Figure 7]. All stimulation groups used a significantly higher explicit component during the horizontal context compared to the vertical context, suggesting that the horizontal context biased all individuals toward using a greater explicit component (HOR: GROUP = 24.79 ± 8.47°, CB = 26.55 ± 8.65°, dlPFC = 25.10 ± 9.91°, SHAM = 22.48 ± 6.81°; VER: GROUP = 14.28 ± 10.26°, CB = 10.15 ± 11.36°, dlPFC = 17.26 ± 6.33°, SHAM = 15.56 ± 11.99°).

## Discussion

Here, we present data from three experiments showing variable effects of anodal tDCS over either the cerebellum or the dlPFC that may lead to reduced target error and improved performance on a visuomotor adaptation task compared to a sham control group. Although it has previously been shown that anodal cerebellar tDCS improves visuomotor adaptation compared to a sham group, this is the first demonstration that anodal left dlPFC tDCS also significantly improves visuomotor adaptation, compared to both cerebellar tDCS and sham groups. However, while we hypothesized that the dlPFC group would reduce target error via a greater explicit component, our results show that this is only a non-significant trend. This suggests anodal dlPFC stimulation may improve visuomotor adaptation, but not solely through increasing the explicit component. We also found that this effect was extremely context-dependent, such that it was only observed when the screen was placed in a horizontal, rather than vertical, orientation, and only with endpoint, rather than online, feedback.

In contrast, and in line with more recent reports, we also found that cerebellar stimulation only weakly, and nonsignificantly, facilitated visuomotor adaptation, and only in a particular context (vertical screen orientation and online feedback). Our effect size was in line with recent reports (~d = 0.6–0.8; Jalali et al., 2017; Oldrati and Schutter, 2017), which showed moderate effects of cerebellar tDCS. However, these findings deviate from the initial strong effects reported in earlier cerebellar tDCS studies (e.g., Galea et al., 2011), and suggests that larger sample sizes are needed to show potentially significant effects. We also found that improvements of target error with cerebellar stimulation were related to greater contributions of implicit mechanisms, but similar to the dlPFC experiment, this effect was variable across individuals and not statistically significant.

Put together, these experiments demonstrate that both dlPFC and cerebellar stimulation may have effects on visuomotor adaptation, but these effects are highly context dependent and variable across individuals. Notably, a major limitation of this study is the small sample sizes per group. As our effect sizes from all of these results are moderate, this suggests that larger sample sizes are needed to confirm these results. However, these results do provide interesting preliminary support for the hypothesis that it may be possible to enhance a motor behavior such as visuomotor adaptation by engaging different neural substrates. Further research is needed to confirm these findings, but if true, this opens interesting opportunities for neurorehabilitation, where engaging an intact mechanism of learning could help compensate for another impaired mechanism.

In addition, we show that dlPFC and cerebellar stimulation can be weakly associated with explicit and implicit mechanisms, respectively, and only in particular contexts. However, as this was not statistically significant in our sample, these results suggest that explicit and implicit components may not be easily or cleanly dissociable and, similarly, that the effects of dlPFC and cerebellar stimulation on subcomponents of visuomotor adaptation may not be easily dissociable. That is, while each may exert a stronger influence on one mechanisms (i.e., dlPFC stimulation on explicit learning and cerebellar stimulation on implicit learning), there are likely also interactions between explicit and implicit learning and these neural regions that vary by individual.

Finally, we note the large interindividual variability across our sample, as evidenced in our individual subject results. This is compounded by a key limitation of our study, which is the relatively small sample sizes for each experiment, which were powered based on results from previous similar studies. However, recent evidence, including this study, is suggesting that larger samples are needed due to high inter-individual variability (Buch et al., 2017; e.g., Lopez-Alonso et al., 2018). Our results suggest that these effects are highly variable across individuals and appear to be driven by a few high performers in the stimulation groups, and low performers in the sham group. This would indicate that stimulation may affect some individuals more than others. This finding of high inter-individual variability following tDCS has been widely discussed in recent years (Ammann et al., 2017; Buch et al., 2017; Lopez-Alonso et al., 2018). Thus, while we discuss the potential implications of our findings, we also urge the reader to use caution in interpreting these results. Our effect sizes from these results are moderate, ranging between d = 0.05–0.9 for most of the significant or trending but not significant results. This suggests that with a larger sample size, a significant effect may be detectable, but this would likely depend heavily on the interindividual variability of each sample. As mentioned in a recent paper by Lopez-Alonso et al. (2018), future studies may consider the role of pre-screening—that is, screening and only including individuals who respond to stimulation over a given region with known effects (e.g., increased cortical excitability following anodal M1 stimulation). The important caveat to this is of course that such a procedure should be clearly documented and reported, and the total number of individuals screened to provide the final sample size should be stated. In addition, visualizing individual subject data, as done here, may be useful for interpreting the strength of any future results. Such findings suggest that stimulation does not have strong effects on everyone, but may have beneficial effects for some.

### Enhancing either implicit or explicit mechanisms could improve adaptation

While previous research maintained that adaptation occurs through primarily implicit means, more recent work has shown that both implicit and explicit processes affect learning (Taylor et al., 2014; Bond and Taylor, 2015; McDougle et al., 2015). Here, we showed that enhancing either mechanism may improve visuomotor adaptation, compared to sham, depending on the context. Specifically, excitatory anodal tDCS over the cerebellum led to slightly greater implicit learning, which could have facilitated target error reduction compared to a sham group in one context (vertical screen with online feedback). Conversely, anodal tDCS over the dlPFC tended to result in a slightly greater reliance on explicit learning mechanisms, reducing target error more than the sham group in a different context (horizontal screen with endpoint feedback). Notably, however, neither of the effects on implicit or explicit mechanisms was statistically significant, suggesting they are not cleanly dissociable. This points to the fact that each mechanism has a role that could potentially be accentuated depending on differing task demands.

The cerebellum has been shown to play a role in visuomotor adaptation (Martin et al., 1996; Wolpert et al., 1998; Maschke et al., 2004; Smith and Shadmehr, 2005; Tseng et al., 2007). Here, we support these findings and demonstrate that the cerebellum may enhance visuomotor adaptation with a stronger effect on implicit learning mechanisms. Interestingly, this effect only becomes evident when tested in a vertical context, which may have biased individuals to rely more on implicit learning. In addition, as shown in previous work, these effects are weak and variable (Jalali et al., 2017).

While there has previously been discussion that the cerebellum may also play a role during the performance of cognitive tasks (Ramnani and Miall, 2001; Pope and Miall, 2012), including explicit learning in a visuomotor rotation task (Butcher et al., 2017), stimulating the cerebellum here did not show a clear impact on explicit learning. However, the cerebellar group, along with the other groups, relied more heavily on explicit learning than implicit learning in the horizontal (but not vertical) condition, as seen in Experiment 3. This suggests that cerebellar stimulation facilitates implicit learning when this mechanism is most needed; however, when another strategy is readily and easily available, stimulating the cerebellum may not provide further benefits.

Previous work has also suggested a role of the dlPFC in explicit motor learning (Anguera et al., 2010; Galea et al., 2010; Taylor and Ivry, 2012). Here, we showed that anodal tDCS over the left dlPFC leads to significantly improved visual motor adaptation, reducing target error. This appears to occur mostly by increasing explicit aiming compared to sham, although the increase in explicit aiming was only a trend and not significant, again likely due to high variability in the sham group. This effect is also context-dependent, and evident only in the horizontal, endpoint feedback condition, which may bias individuals to rely on explicit strategies. This finding is important because previous research has shown that when individuals are given a strategy (e.g., “aim 45° counterclockwise”), their initial improvements in motor learning result into greater errors over time, as implicit mechanisms come into play (Mazzoni and Krakauer, 2006). Here, however, we make an important distinction between giving individuals a strategy (e.g., “aim 45° counterclockwise”) and biasing them toward utilizing strategy-based learning through excitatory stimulation over the dlPFC. The latter allows them to both develop and *adapt* an explicit strategy to accommodate changes in hand position due to the implicit component's increasing role over time (e.g., initially aim 45° counterclockwise, but then reduce this to 40°, 35°, and so forth intelligently).

Importantly, however, the lack of consistently significant results indicates that there may not be a clean dissociation between the cerebellum and dlPFC. This is also evident in the fact that some of the strongest results emerged in direct comparisons between each active site and sham, vs. in comparisons between the two active conditions directly. It is likely that because stimulation of either dlPFC or cerebellum can lead to benefits in visuomotor adaptation performance, the differences between stimulating the two are not as remarkable as the differences between active stimulation of either region and no stimulation at all. In addition, although each one seems to exert a stronger effect on implicit and explicit learning, respectively, stimulation of each region may also influence the other region, and its subsequent function. Previous studies have shown both functional and structural connections between these regions (Strick et al., 2009; Krienen and Buckner, 2010), suggesting they may be part of the same network. Thus, while there are some trends suggesting differences from stimulating each region, there is likely also considerable interplay between these two regions. Future research might explore whether the effects of stimulation are related to the individual's baseline connectivity between these two regions.

### Context affects reliance on implicit vs. explicit learning mechanisms

Our study also demonstrates strong context-dependent effects in both target error and the relative contributions of implicit vs. explicit components. Previous research has shown that online feedback engages a greater implicit component, while endpoint feedback utilizes a greater explicit, cognitive component (Hinder et al., 2008; Taylor et al., 2014). In addition, different contextual aspects of visuomotor adaptation can affect learning (Ghilardi et al., 1995; Pine et al., 1996; Jalali et al., 2017). Our results extend these findings, showing that modifications of the visuomotor transformation between hand space and visual feedback (horizontal vs. vertical screens) also changes the relative contributions of implicit and explicit mechanisms—as well as how stimulation affects these mechanisms.

Looking first at the sham group, we found that a vertical context, with a 90° visuomotor transformation from the cursor display to the hand, leads to worse performance on visuomotor adaptation when compared to a horizontal context (1-to-1 mapping). This is a consistent, significant effect across groups. In other words, it becomes more difficult to adapt with the added visuomotor transformation. In addition, the data suggest that in some cases, the vertical context tends to decrease the explicit component while maintaining the same amount of implicit learning. This may be due to the fact that the explicit component requires a conscious, cognitive strategy that is more taxed when performing trial-by-trial calculations with the addition of a visuomotor transformation. On the other hand, in the horizontal setting, with a one-to-one mapping of one's hand position onto the visual feedback, developing a cognitive strategy might be more straightforward. The 3 × 2 (group by context) ANOVA also reinforces the idea that context (horizontal vs. vertical screen) is a driving force across all stimulation groups, with all groups performing worse and using more implicit learning in the vertical context, and performing better and using more explicit learning in the horizontal context. The intersubject variability of these effects also suggests that individuals may use varying combinations of implicit and explicit components depending on task demands, which is an area warranting future investigation.

### The role of the left dLPFC in motor learning

Our results finally suggest a novel role that the left dlPFC may play in visuomotor adaptation, and also potentially in explicit learning, particularly in contexts that bias the individuals toward using a cognitive strategy. These effects are not surprising given the wide breadth of literature that has explored the role of the dlPFC in memory and other higher-level cognitive functions (Waltz et al., 1999; Kroger et al., 2002); however, there have been few studies explicitly linking anodal dlPFC stimulation to improvements in motor learning (Anguera et al., 2010). Our results build on this work, showing a causal link between left dlPFC stimulation and visuomotor adaptation, as well as showing a weaker relationship between left dlPFC stimulation and explicit learning. However, these findings open up new questions, such as whether there are differences in the roles of the left and right dlPFC during visuomotor adaptation, and whether/how these are functionally connected to other cognitive networks or other motor regions to influence learning (Ramnani and Miall, 2001; e.g., Middleton and Strick, 2001). While the left dlPFC now represents a potential target for improving explicit motor learning, it is likely only part of a more complex neural network of regions that influence these high-level cognitive processes during motor adaptation.

## Conclusion

Overall, our results indicate that both the cerebellum and dlPFC may play roles in visuomotor adaptation, with the former more strongly influencing implicit mechanisms and the latter more strongly influencing explicit aspects of learning. We also show important context-dependent differences in how these mechanisms and regions are engaged. Finally, we note that these results are highly influenced by interindividual variability, and that the effects of brain stimulation do not seem to be consistent across individuals. Indeed, some results may be driven by poor performers in the sham group, although it is not clear whether these poor performers might have benefitted from brain stimulation themselves. Overall, although further research is needed, these results provide preliminary evidence that suggests that tDCS of the cerebellum and the dlPFC may influence visuomotor adaptation.

## Author contributions

S-LL designed the study, collected data, analyzed data, and wrote the manuscript. TT and JR collected study data and contributed to the manuscript. TT also created illustrations used in Figure 1. PB and JT contributed to the study design and experimental paradigm as well as analyses, and provided feedback on the manuscript. PC designed the study and provided feedback on the manuscript.

### Conflict of interest statement

The authors declare that the research was conducted in the absence of any commercial or financial relationships that could be construed as a potential conflict of interest. The reviewer ESC and handling Editor declared their shared affiliation at the time of the review.
